# Cognitive reappraisal training as an intervention in patients with functional movement disorders: a placebo-controlled EEG pilot study

**DOI:** 10.1007/s00702-025-03028-5

**Published:** 2026-01-24

**Authors:** Melanie Suette, Petra Schwingenschuh, Daniela Kern, Birgit Ludwig, Lukas Gattermeyer-Kell, Mariella Kögl, Maria Zangl, Corinna M. Perchtold-Stefan

**Affiliations:** 1 Department of Neurology, Medical University of Graz, Auenbruggerplatz 22, 8036 Graz, Austria; 2 Department of Psychology, University of Graz, Graz, Austria; 3 Department of Neurology, Medical University of Vienna, Vienna, Austria

## Abstract

Functional movement disorders (FMD) are common and disabling neurological conditions characterized by impaired voluntary motor control in the absence of structural disease. Accumulating evidence suggests that emotion regulation deficits contribute to the pathophysiology of FMD. This randomized, placebo-controlled pilot study investigated the clinical and neural effects of a digital cognitive reappraisal training program in patients with FMD, targeting emotion regulation as a potential therapeutic mechanism. Twenty adult patients diagnosed with clinically definite FMD (mean age = 50.7 years, 11 females) were randomly assigned in a 1:1 ratio to either an intervention group practicing cognitive reappraisal (*n* = 11) or an active control group completing reflection exercises (*n* = 9). Both groups underwent 14 brief, app-based training sessions over four weeks. Clinical outcomes were assessed before and after the intervention using standardized measures of mood, motor function, and overall illness severity. Simultaneously, brain activity at rest and during a standardized reappraisal task was recorded via electroencephalography, focusing on prefrontal alpha asymmetry as a neural marker of reappraisal engagement. Compared to the control group, the intervention group showed significantly greater improvements in mood (Mdiff = 0.542, *p* = .010), motor function (Mdiff = 0.542, *p* = .033), and overall illness severity (Mdiff = 0.528, *p* = .040). Both groups improved over time in mood (*p* = .030), tremor severity (*p* = .014), and subjectively rated motor function (*p* = .022), suggesting non-specific benefits of structured emotional self-reflection. Crucially, electroencephalography data revealed a significant post-training increase in right-lateralized frontopolar resting-state activity in the intervention group (Mdiff = 13.29, *p* = .026), aligning with prior evidence linking right prefrontal activity to successful reappraisal. Although task-related EEG activity did not differ significantly between groups, a statistical trend (*p* = .115, d = 1.14) suggests increased right frontal activity during reappraisal generation in the intervention group. Reappraisal training performance correlated with EEG changes and with the frequent use of the distancing strategy “relativizing,” implicating right prefrontal involvement in specific cognitive control processes. These findings provide preliminary evidence that cognitive reappraisal training delivered via mobile app is feasible and may enhance both clinical outcomes and prefrontal brain function in FMD. Future studies with larger samples are needed to confirm these effects and better understand the neural mechanisms of digital interventions for emotion regulation in functional neurological disorders.

## Introduction

Functional Movement Disorders (FMD) are prevalent neurological conditions characterized by positive clinical signs (e.g. inconsistency, distractibility, and entrainability) that are inconsistent and incongruent with other movement disorders. FMDs significantly impair patients’ quality of life, with functional tremor patients experiencing disability comparable to Parkinson’s disease (Zeuner and Sidiropoulos 2019).

Research suggests that emotion regulation (ER) deficits play a role in FMD, with patients with functional neurological symptoms (FNS) showing more frequent use of maladaptive emotion regulation strategies like emotion suppression and less frequent use of adaptive strategies like cognitive reappraisal compared to healthy controls (Schnabel et al. 2022). A functional imaging study showed that emotion regulation tasks in patients with functional neurological disorders (FND) shift neuromagnetic activity from frontocortical to sensorimotor regions, reflecting deficient emotion regulation and worsening functional neurological symptoms (Fiess et al. 2015). Similarly, altered activation and connectivity in emotion-processing networks have been observed in individuals with functional tremors (Espay et al. 2018). Functional imaging research has also identified altered resting-state activity in the right temporoparietal junction (rTPJ) in patients with FMD, which the authors linked to a reduced sense of self-agency (Maurer et al. 2016).

Apart from temporoparietal junction hypoactivity, brain imaging studies suggest a frontal dysfunction (e.g., dorsolateral prefrontal cortex) in regulating emotions as well as abnormal limbic function in association to motor or sensory networks to be characteristic for FND patients (Aybek and Vuilleumier 2016). In a fMRI study with an avoidance learning task, patients with FND showed decreased left dorsolateral prefrontal cortex (DLPFC) and increased bilateral amygdala responses to negative feedback (monetary loss) compared to healthy controls (Morris et al. 2017). Overall, these studies suggest deficient emotional processing and emotion regulation in patients with FND and might therefore represent suitable targets for therapeutic interventions.

To date, there are no guidelines on treatment of FMD, but there is evidence for benefits from treatments such as specialized physiotherapy, cognitive behavioral therapy, and psychodynamic therapies. Still, access to these treatments is often difficult when balancing costs, time, and accessibility (Schwingenschuh 2022; Serranová et al. 2024; Sharma et al., 2016, Dallocchio et al. 2016). However, new, digital, remote, and more personalized treatment approaches are emerging, which may also help to close this treatment gap with regard to emotion regulation (Lin and Espay 2021).

A promising approach to reduce symptomatic burden and increase quality of life in patients is training them to practice adaptive strategies like cognitive reappraisal in daily life (Kleinstäuber et al., 2019). Cognitive reappraisal refers to deliberately viewing an emotionally evocative event from a different perspective and reinterpreting its meaning, thereby changing its emotional impact (Gross and John 2003). Numerous behavioral studies have demonstrated that more frequent use of reappraisal in everyday life is associated with reduced depressive symptoms, lower levels of stress- and anxiety-related psychopathology, and enhanced cognitive and social functioning (see Riepenhausen et al. 2022). Moreover, brief, well-structured cognitive reappraisal trainings have shown potential as practical, accessible methods for improving quality of life, even for individuals facing high symptom burden (Denny & Ochsner, 2014; Perchtold-Stefan et al. 2024).

For this reason, the present study investigated the clinical and neural effects of a digital cognitive reappraisal training program in patients with FMD, to see if this training can improve emotion regulation skills and underlying brain mechanisms, while simultaneously reducing symptomatic burden. Cognitive reappraisal generation is widely linked to neural changes, particularly in prefrontal alpha activation patterns measurable with electroencephalography (EEG). In this regard, prefrontal EEG-alpha asymmetry has emerged as a key marker of reappraisal performance: Previous EEG studies reported that either more left-lateralized or more right-lateralized prefrontal cortex activity during cognitive reappraisal generation was linked to greater reappraisal success and active coping with stress and anxiety (Papousek et al. 2017; Perchtold et al. 2018, 2019; Perchtold-Stefan et al., 2024). Additionally, a short cognitive reappraisal EEG intervention was successfully implemented and validated in a student sample suffering from anxiety, who showed adaptive changes in prefrontal EEG-alpha asymmetry – underlining more adaptive coping with anxiety – post-intervention (Perchtold-Stefan et al. 2024). Notwithstanding the mixed findings with regard to the direction of prefrontal lateralization, lateralized prefrontal cortex activity in relation to cognitive reappraisal may be an informative marker for testing the effectiveness of emotion regulation interventions in patients. As frontal dysfunction and impaired emotional regulation play a role in the pathophysiology of FND, we thus expected EEG alpha asymmetry changes for prefrontal positions in particular (hypothesis-driven analysis). However, as other brain imaging studies have also found temporal and parietal brain regions involved in the cognitive reappraisal process (see Picó-Pérez et al., 2017), we also extended our analyses to other cortical regions (central, temporal, parietal, occipital; exploratory analysis).

Altogether, this placebo-controlled pilot study explores whether app-based cognitive reappraisal training enhances FMD patients’ well-being and induces measurable neural changes, particularly in prefrontal EEG patterns during cognitive reappraisal.

## Materials and methods

A total of 26 patients with clinically definite functional movement disorders were recruited between June 2023 and April 2024; 18 completed the study, while 8 dropped out for personal reasons. Inclusion criteria were age 18–80 and no contraindications for EEG (e.g., epilepsy, brain surgery) and a clinically definite FMD according to diagnostic criteria (Gupta and Lang, 2009) and best practice recommendations (Espay et al. 2018). Telephone follow-up took place 14 days after baseline, clinical follow-up after 4 weeks. As a pilot study, no formal sample size calculation was conducted; the sample size was based on feasibility and prior pilot work.

**Fig. 1:**
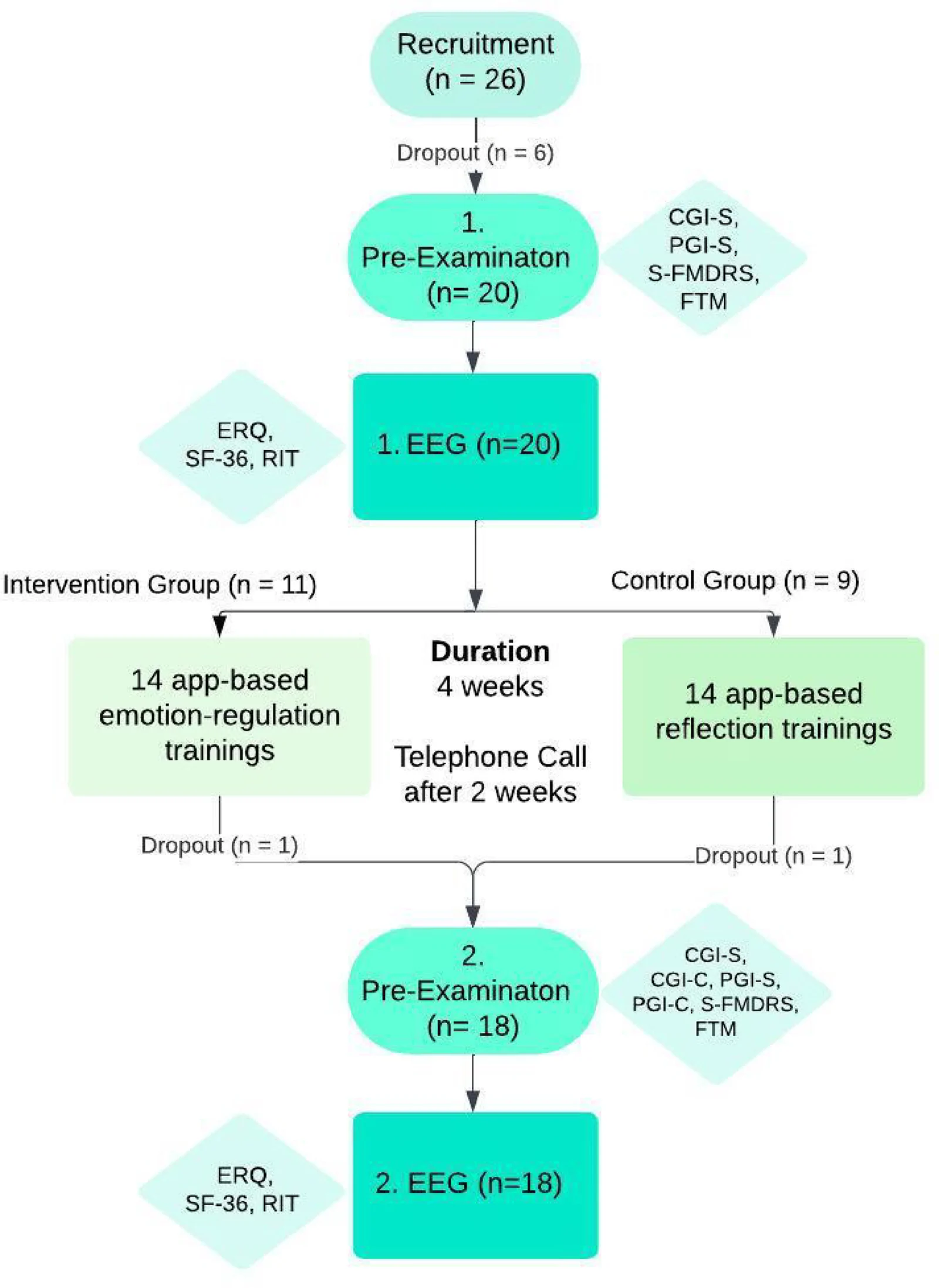
Flowchart on the study design and utilized questionnaires. Clinical/Patient Global Impression – Severity/Change (CGI-S, CGI-C, PGI-S, PGI-C): A 5-point scale assessing the clinical impression of disease severity/change, completed by medical professionals/patients (Busner and Targum 2007; Guy 1976) – for the purpose of this study, three items have been created regarding mood, motor function and overall disease; Simplified Version of the Psychogenic Movement Disorders Rating Scale (S-FMDRS): A scale assessing various aspects of functional movement disorders, including movement quality, symptom distribution, severity, and daily life impairment (Nielsen et al. 2017); Fahn-Tolosa-Marin Tremor Rating Scale (FTM): A Scale assessing tremor severity and related motor impairment (Fahn et al. 1993); Reappraisal Inventiveness Test (RIT): A test evaluating the ability to reframe emotionally distressing situations (e.g., anger or fear) to reduce their emotional impact (Weber et al. 2014; Papousek et al. 2017; Perchtold et al. 2018, 2019); Emotion Regulation Questionnaire (ERQ): A questionnaire assessing emotion regulation strategies, including reappraisal and suppression (Abler and Kessler 2011); Level of Personality Functioning Scale - Brief Form (LPFS-BF): A short self-report questionnaire evaluating personality functioning across self- and interpersonal domains (Weekers et al. 2019); SF-36 (Short Form Health Survey − 36) A self-report questionnaire assessing health-related quality of life across 8 dimensions, including “General Health” and “Emotional Well-Being” (Brazier 1995).66

All patients underwent a detailed clinical examination at the Department of Neurology, Medical University of Graz, including the Fahn-Tolosa-Marin Clinical Rating Scale for Tremor (FTM), the Simplified Version of the Psychogenic Movement Disorders Rating Scale (S-FMDRS), and the clinical (CGI-S) and the patient (PGI-S) global impression of symptom severity/change scales (CGI-S, PGI-S, CGI-C, PGI-C) before and after the intervention (see Fig. 1). Clinician- and patient-rated versions were clearly distinguished throughout, with CGI scales completed by the investigator and PGI scales by the patients themselves.

 Additionally, patients were asked during the telephone visit and the 2nd clinical visit if any adverse events had occurred. No harms were expected or systematically assessed due to the non-invasive nature of the intervention.

A placebo-controlled design was used to control for expectancy effects and nonspecific intervention factors. We also attempted double-blinding with our approach: Both groups received structurally similar app-based training tasks with identical duration and format, while participants as well as the physicians conducting the clinical ratings were unaware of group allocation. Participants were randomly assigned to the intervention or placebo group in a 1:1 ratio using simple randomization without blocking or stratification. Allocation was determined by drawing sealed, pre-labelled paper slips from a container, which were thoroughly mixed before each draw.

Although the participants were aware of the nature of their assigned training tasks (cognitive reappraisal vs. reflection), participants were not informed about the study hypotheses or which intervention was considered the “active” treatment, minimizing expectation bias. Full debriefing regarding group assignment occurred only after study completion. Thus, participants were blinded to the experimental condition throughout the intervention phase.

The lead experimenter (MS), who administered the EEG, RIT, SF-36, and ERQ assessments, was informed of group allocation for practical reasons. However, all clinical outcome ratings—including the FTM, S-FMDRS, PGI-C, PGI-S, CGI-C, and CGI-S—were performed by independent clinicians who were fully blinded to both group allocation and training content, thereby ensuring objectivity in the assessment of primary endpoints. Neither the rating clinicians nor the patients were aware of group assignments at any point during the study. The interventions were matched in format, duration, and delivery to ensure comparable user engagement across groups.

Finally, both training programs were matched for duration, mode of delivery, and overall user engagement, serving to equalize non-specific effects such as attention, time, and perceived support. Thus, while traditional double-blinding (in the pharmacological sense) was not feasible due to the nature of the interventions, the study design incorporated robust blinding procedures where possible and appropriate for a psychological intervention trial.

The following Flowchart in Fig. 1 gives an overview on the study design aswell as the questionnaires used (also see Supplemental material/methods). In the EEG laboratory, participants first completed questionnaires, followed by EEGmontage.

### EEG procedure and cognitive reappraisal task

Brain activation at rest and during cognitive reappraisal generation was measured with EEG, and analyzed with regard to prefrontal EEG alpha asymmetry, indicating different activation patterns in the left and right prefrontal cortex, which emerged as a robust EEG correlate of cognitive reappraisal generation in previous studies (Papousek et al. 2017; Perchtold et al. 2018; Perchtold-Stefan et al. 2024).

A 2-minute resting EEG measurement was conducted while participants calmly focused on a green dot on the screen. They were then equipped with a chest-mounted microphone and introduced to the Reappraisal Inventiveness Test (RIT, Weber et al. 2014; Perchtold-Stefan et al., 2024) — a standardized task designed to assess individuals’ ability to flexibly generate cognitive reappraisals, i.e. alternative interpretations of emotionally distressing situations that help reduce their emotional impact. For the RIT, participants received written and verbal instructions, including a brief keyboard practice. In the RIT, they were presented with two anxiety-inducing and two anger-inducing scenarios (see SM for examples), each shown on the screen for 20 s and separated by a 5-second fixation cross. Participants were instructed to imagine themselves in each provided scenario and then, once a white question mark turned green upon pressing the space bar, to verbally generate as many different reappraisal strategies as possible — such as reframing the situation, altering its meaning, or focusing on positive aspects. They had up to three minutes per scenario and ended their response by pressing the space bar again. Participants answers were recorded via a microphone, transcribed, and subsequently analysed. After each scenario, participants rated how much anxiety or anger it had induced on a 6-point Likert scale. Two versions of the RIT (using different items) were administered during EEG recording, with version A given to half the subjects during the first appointment and version B during the second, and vice versa for the other half.

The administration of the RIT during EEG ensures that participants actively try to engage in cognitive reappraisal (and not any other emotion regulation strategy). As a quantitative metric of cognitive reappraisal success, we quantified RIT fluency, which is the total number of generated unique reappraisal ideas (see Papousek et al. 2017; Perchtold et al. 2018, 2020). This metric serves as an established indicator of successful cognitive reappraisal engagement, and measures individual differences in fluent and flexible cognitive reappraisal generation (see Papousek et al. 2017; Perchtold et al. 2018, 2019). RIT Fluency was determined by two independent raters (ICC two-way mixed, consistency, pre-test: 0.94; post-test: 0.87). However, as some cognitive reappraisal strategies may be more effective than others, RIT answers were also coded for the reappraisal strategies of problem-solving (ICC two-way mixed pre-test: 0.87; post-test: 0.80), positive re-interpretation (ICC two-way mixed pre-test: 0.79; post-test: 0.77), and relativizing (ICC two-way mixed pre-test: 0.71; post-test: 0.88) that reflect different qualities of reappraisal ideas (see Perchtold-Stefan et al.,^2024^).

### Participant introduction to training

Finally, the training introduction differed between the intervention and control group (see Supplementary Material for full protocol). The intervention group received a brief theoretical overview of cognitive reappraisal and was then guided through 14 daily app-based training sessions over a period of two weeks. Each session lasted approximately three minutes and involved emotionally evocative scenarios targeting fear or anger. Participants were instructed to generate as many alternative, less distressing thoughts as possible to reduce the emotional impact of the situation—mirroring the structure of the Reappraisal Inventiveness Test (RIT). For example, one scenario described walking home alone at night while being followed, prompting participants to reinterpret the situation in ways that would reduce anxiety (e.g., “Maybe the person lives nearby and is heading in the same direction”). Another anger-related scenario involved discovering that a friend had neglected to water one’s plants during a vacation. In contrast, the control group received an introduction to general reflection techniques and completed matched sessions in which they responded to neutral prompts without deliberate emotion regulation. These included tasks such as “What special thing happened today that you would like to record?” or “Think of a small, unexpected, positive event.” Both trainings were matched in frequency, duration, and format to control for expectancy effects and general engagement, allowing us to isolate the specific effects of cognitive reappraisal practice.

App data was analyzed to determine compliance with training. Over the 14-day intervention period, participants in the intervention group completed an average of 11.82 cognitive reappraisal responses (SD = 3.25, Min = 6, Max = 14), while those in the placebo group completed an average of 10.22 reflection responses (SD = 3.60, Min = 5, Max = 14).

### EEG metrics

EEG data were recorded with a Brainvision ActiCHamp Plus Research Amplifier (1000 Hz sampling rate, low cutoff DC, high cutoff 280 Hz) in a shielded room. Nineteen active electrodes (FP1, FP2, F3, F7, FZ, F4, F8, C3, CZ, C4, T7, T8, P3, PZ, P4, P7, P8, O1, O2) were positioned according to the international 10–20 system. The ground electrode was placed on the forehead and EEG was referenced to the nose and re-referenced offline using an average ear reference (see Perchtold-Stefan et al., 2024). Electrode impedances were kept below 30 kΩ for EEG electrodes and below 10 kΩ for ground and reference electrodes.

EEG pre-processing included removing low-frequency drifts (1 Hz filter), setting a notch filter (50 Hz), referencing to an averaged ear reference, and removing intervals that indicated ocular or muscle artifacts by visual inspection. Only intervals in which participants generated reappraisal ideas were analysed (i.e., idea generation), excluding reading, speaking, and rating intervals. Analyses focused on the EEG alpha band (8–12 Hz).

Laterality coefficients (LQ) were calculated as LQ = [(L + R)/(L - R)] × 100, where R is the right and L the left alpha power. Eight LQs were derived for frontopolar (Fp1/Fp2), dorsolateral frontal (F3/F4), ventrolateral frontal (F7/F8), central (C3/C4), temporal (T7/T8), parietal (P3/P4), ventrolateral parietal (P7/P8), and occipital (O1/O2) regions. Positive values indicate more left-hemisphere alpha power, reflecting higher right-hemisphere cortical activity due to the inverse relationship between alpha power and brain activity (see Papousek et al. 2017).

## Statistical analysis

Statistical analyses were conducted using IBM SPSS Statistics (version 29) with an alpha level of 0.05. Mixed ANOVAs and paired-sample T-tests analyzed group differences across time points, while Pearson’s correlations assessed relationships between variables. Moreover, exploratory correlation analyses between EEG changes and training performance were conducted. Missing data were handled using pairwise exclusion. No imputation was performed.

### Standard protocol approvals, registrations and patient consents

The study protocol was reviewed and accepted by the institutional review board of the Medical University of Graz. All patients gave written informed consent to participate in the study. The study was prospectively registered on ClinicalTrials.gov (35–315 ex 22/23).

## Results

### Main outcome

As shown by the absolute frequencies in Table 1, patients in the control group did not differ substantially from the intervention group with respect to non-motor symptoms, including neurological and psychiatric comorbidities. Likewise, no significant group differences were observed in emotional well-being, pain, or general health, as assessed by the SF-36, nor in emotion regulation strategies (reappraisal and suppression), as measured by the ERQ (see Table 2).

**Table 1:** gives an overview on functional diagnosis and symptoms as well as neurologic and psychiatric comorbidities of patients per group

Characteristic	Total (*n* = 20)	Intervention Group (*n* = 11)	Control Group (*n* = 9)
Functional neurological symptoms			
Functional limb tremor	11	6	5
Functional head tremor	2	0	2
Functional palatal tremor	1	1	0
Paroxysmal movement disorder	2	1	1
Functional gait disturbance	1	0	1
Abdominal myoclonus	1	1	0
Functional craniocervical dystonia	1	1	0
Mild functional paralysis	1	1	0
Functional comorbidities			
Irritable bowel syndrome	3	0	3
Functional/dissociative seizures	1	1	0
Chronic fatigue syndrome	1	0	1
Fibromyalgia	1	1	0
Neurologic comorbidities			
Young onset Parkinson’s disease	1	1	0
Restless Leg Syndrome	1	1	0
Migraine with aura	1	1	0
Psychiatric comorbidities			
Depression	8	5¹	3
Anxiety disorder	2	0	2
PTSD	1	1	0
Chronic insomnia	1	1	0
Somatic comorbidities			
Psoriasis	1	1	0
Mb. Meulengracht	1	1	0
Hashimoto thyroiditis	1	0	1
Arterial hypertension	1	0	1
Mb. Bechterew	1	0	1
Hyperlipidaemia	1	1	0
Hypothyroidism	2	2	0
Nicotine abuse	2	1	1
Neurodermatitis	1	1	0
Chronic kidney insufficiency	1	1	0

¹ One of these patients dropped out after the first visit due to an acute depressive episode.

**Table 2:** Main outcome

Parameter	Total (*n* = 20)		CG (*n* = 9)		IG (*n* = 11)		Difference
	M	SD	M	SD	M	SD	
Age [years]	50.70	13.94	52.6	5.19	49.09	3.92	*M*_diff_ = −3.58, *p* =.58
S-FMDRS			8.22	7.68	7.64	4.52	*M*_diff_ = − 0.59, *p* =.834
FTM A			8.55	5.81	5.3	5.56	*M*_diff_ = −3.26, *p* =.229
FTM B			4.33	4.92	3.80	3.39	*M*_diff_ = − 0.53, *p* =.785
FTM C			4.33	4.92	3.80	3.39	*M*_diff_ = − 0.53, *p* =.785
CGI-S (mood)			1.89	1.17	2.10	0.57	*M*_diff_ = 0.21, *p* =.632
CGI-S (motor function)			2.11	0.93	2.00	0.47	*M*_diff_ = −0.11, *p* =.752
CGI-S (general change)			1.67	0.50	1.90	0.32	*M*_diff_ = 0.23, *p* =.236
PGI-S (mood)			2.33	1.41	2.40	1.43	*M*_diff_ = 0.07, *p* =.920
PGI-S (motor function)			2.67	1.23	2.60	1.17	*M*_diff_ = 0.07, *p* =.905
PGI-S (general change)			2.22	1.20	2.10	0.88	*M*_diff_ = −0.12, *p* =.802
ERQ subscale reappraisal			70.22	16.26	64.36	16.73	*M*_diff_ = −0.11, *p* =.800
ERQ subscale suppression			3.53	1.51	3.32	1.84	*M*_diff_ = −0.21, *p* =.787
SF-36 subscale emotional well-being			70.22	16.26	64.36	16.73	*M*_diff_ = −8.86, *p* =.440
SF-36 subscale pain			53.89	35.64	48.18	37.41	*M*_diff_ = −5.71, *p* =.733
SF-36 subscale gen. health			58.74	28.49	58.50	18.57	*M*_diff_ = −0.24, *p* =.983
LQ frontopolar Rest			0.94	6.27	−2.08	5.42	*M*_diff_ = −3.02, *p* =.354
LQ dorsolateral-frontal Rest			−1.25	6.34	0.27	7.94	*M*_diff_ = 1.53, *p* =.671
LQ ventrolateral-frontal Rest			−1.73	15.58	−1.34	16.00	*M*_diff_ = 0.38, *p* =.961
LQ zentral Rest			1.30	10.01	3.43	10.79	*M*_diff_ = 2.13, *p* =.681
LQ temporal Rest			4.24	7.72	−0.61	18.78	*M*_diff_ = −4.85, *p* =.604
LQ dorsolateral-parietal Rest			−0.91	5.61	5.85	11.42	*M*_diff_ = 6.77, *p* =.150
LQ ventrolat.-parietal Rest			−2.82	6.70	10.06	29.84	*M*_diff_ = 12.88, *p* =.253
LQ occipital Rest			4.99	9.34	9.79	11.28	*M*_diff_ = 4.80, *p* =.427
LQ frontopolar RIT			1.81	6.81	−4.17	9.04	*M*_diff_ = −5.98, *p* =.242
LQ dorsolateral-frontal RIT			−3.89	5.03	−1.99	7.42	*M*_diff_ = 1.91, *p* =.566
LQ ventrolateral-frontal RIT			−4.87	19.86	2.59	18.78	*M*_diff_ = 7.45, *p* =.453
LQ zentral RIT			−2.13	13.66	1.13	7.86	*M*_diff_ = 3.26, *p* =.550
LQ temporal RIT			−2.41	4.60	2.90	17.90	*M*_diff_ = 5.30, *p* =.495
LQ dorsolateral-parietal RIT			−3.40	7.67	3.55	15.23	*M*_diff_ = 6.95, *p* =.263
LQ ventrolateral-parietal RIT			−7.36	24.41	6.75	26.33	*M*_diff_ = 14.11, *p* =.304
LQ occipital RIT			−1.22	9.47	13.05	12.49	*M*_diff_ = 14.27, *p* =.066

	Parameter	CG			IG				CG		IG			
		M	SD		M	SD			M	SD	M	SD		
Group effects	CGI-C (mood)								4.13	0.35	4.67	0.50	*t* (14.34) = 2.55, *p* =.010, cohen’s d = 0.44, *M*_diff=_ 0.542, CI[0.088–0.995]	
	CGI-C (motor function)								4.13	0.35	4.67	0.71	*t* (12.04) = 2.03, *p* =.033, cohen’s d = 0.57, *M*_diff=_ 0.542, CI[−0.039–1.123]	
	CGI-C (general change)								4.25	0.46	4.78	0.67	*t (*15) = 1.87, *p* =.040, cohen’s d = 0.58, *M*_diff=_ 0.528, CI[−0.063–1.119]	
Time effects	CGI-S (mood)	2.00	1.20		2.25	0.46			1.75	1.04	1.63	0.74	*F* (1, 14) = 5.81, *p* =.030, *ηp²* = 0.29, *M*_diff_ = 0.438, CI[0.048–0.827]	
	PGI-C (general change)	3.63	0.52		3.78	1.30			4.00	0.00	4.33	0.50	*F* (1, 15) = 3.04, *p* =.102, *ηp²* = 0.17, *M*_diff=_ − 0.465, CI[−1.034–0.104]	
	PGI-C (motor function)	3.50	0.53		3.33	1.41			4.00	0.00	4.22	0.44	*F* (1, 15) = 6.49, *p* =.022, *ηp²* = 0.30, *M*_diff=_ −0.694, CI[−1.276 – −0.113]	

Parital eta squared (,ηp²) is reported as a measure of effect size for ANOVA results. Values of .01, .06, and .14 are commonly interpreted as small, medium and large effects, respectively.

At the first measurement point, the Emotion Regulation Questionnaire (ERQ) and the Short Form Health Survey 36 (SF-36) revealed the following scores. For the ERQ, reassessment values ranged from 2.67 to 6.50 (M = 4.85, SD = 0.94), and suppression scores ranged from 1.00 to 6.25 (M = 4.41, SD = 1.66).

The SF-36 assessment showed that physical functioning ranged from 40.00 to 100.00 (M = 75.31, SD = 20.85). Role limitations due to physical health ranged from 0.00 to 100.00 (M = 45.59, SD = 41.68), while emotional role limitations ranged from 0.00 to 100.00 (M = 57.41, SD = 43.99). Energy levels ranged from 0.00 to 85.00 (M = 43.00, SD = 25.77), and emotional well-being ranged from 40.00 to 96.00 (M = 67.00, SD = 16.36). Social functioning scores ranged from 12.50 to 100.00 (M = 74.34, SD = 29.31). Pain scores were between 0.00 and 100.00 (M = 50.75, SD = 35.78), and general health ranged from 11.20 to 85.00 (M = 58.60, SD = 22.32).

The results indicate that the IG showed a greater improvement in mood, motor daily functioning and overall disease progression compared to the CG, as well as a trend toward greater subjectively reported overall disease change. Both groups demonstrated significant improvements in tremor severity and marginally significant improvements in FMD symptoms as well as betterments in the impact of the disease on mood and physical functioning. Additionally, there was a trend toward improvement in the subjectively evaluated overall disease progression in both groups.

Significant results are displayed in Table 2. No significant group or time effects could be found in any other questionnaires or brain regions (also see Supplemental material/results).

**Fig. 2:**
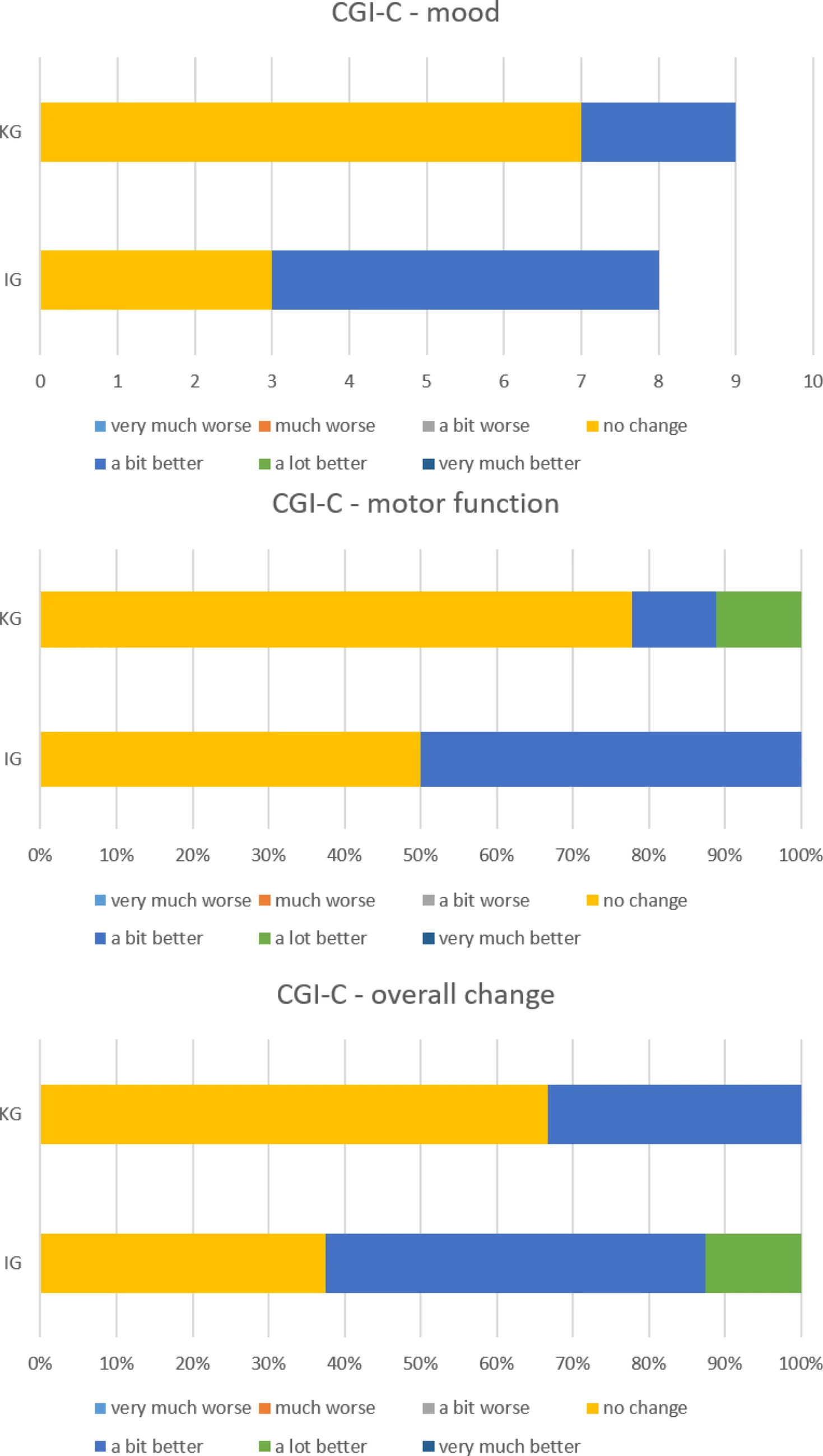
Main outcome CGI-C at second time point, data were analysed using an independent samples t-test (IG n = 9; CG n = 9), test statistics can be seen in Table 2

Figure 2 provides an overview of the main outcome measure, the Clinical Global Impression of Change, showing the distribution of ratings for mood, motor function, and overall change at the second time point for both groups.

Despite no significant change in the LQ-CC in frontopolar areas during the RIT-task, large effect sizes were observed (T(8) = 1.77, *p* =.115, Cohen’s d = 1.14, CI[1.960–24.620], Mdiff = 6.29, CI[−0.269–2.490], CG: *n* = 6; IG: *n* = 4).

A positive correlation was found between resting-state LQ-CC in frontopolar areas and the reappraisal strategy “relativizing” (*r* =.632, *p* =.037). Additionally, general app-training performance (across both groups) correlated with resting-state LQ-CC in frontopolar areas (*r* =.693, *p* =.018), linking training to brain activity changes. The total number of reappraisals during app training correlated with RIT Fluency at the second measurement point (*N* = 10, *r* =.80, *p* =.005).

Finally, reappraisal performance (RIT Fluency) at the first measurement was positively associated with more right-lateralized dorsolateral frontal activity at rest (*N* = 15, *r* =.63, *p* =.012), linking higher reappraisal performance to right dorsolateral prefrontal cortex function Table 3.

**Table 3:** Secondary outcome

	Parameter	Measurement 1				Measurement 2				
		CG n = 9		IG n = 11		CG = 10		IG = 8		
		M	SD	M	SD	M	SD	M	SD	
Time effects	S-FMDRS	8.63	8.11	7.70	4.76	7.50	6.82	5.40	5.40	*F*(1,16)=3.58,*p*=.077,*ηp² *= 0.18,*M*_diff=_1.713, CI[-0.206 -3.631]
	FTM A	8.75	6.18	5.89	5.56	7.38	5.26	3.56	3.84	*F(1,15)=7.67,p=.014,ηp²*=0.338,*M*_diff=_-1.854, CI[0.427 – 3.281]

		CG n = 6				IG n = 5				
		M		SD		M		SD		
Group effects	LQ CC in frontopolar areas RS	-5.33		6.66		7.96		9.93		*T(9) = 2.65, p* = .026, cohen’s d = 1.61 CI [0.181 – 2.970],* M*_diff_ = 13.29, CI[1.960 – 24.620]

Parital eta squared (,ηp²) is reported as a measure of effect size for ANOVA results. Values of .01, .06, and .14 are commonly interpreted as small, medium and large effects, respectively.

## Discussion

This study compared the neural and clinical outcomes of a 14-day app-based cognitive reappraisal training in patients with FMD to those of a control group who engaged in reflective exercises.

The intervention group showed greater improvement in objectively measured mood, motor function and general illness compared to the control group. Additionally, objectively measured mood as well as subjectively measured motor function and general illness improved over time in both groups, highlighting the effectivity of both interventions.

EEG-alpha asymmetry analysis revealed a significant increase in right-lateralized activity in frontopolar areas during the resting-state measurement for patients in the intervention group. However, no significant changes in EEG-alpha activity in frontopolar areas were observed during cognitive reappraisal generation. Still, as detailed in the results section, a statistical trend also suggests increased right-lateralized activity in frontopolar areas during reappraisal engagement (see Perchtold-Stefan et al., 2024), which was underlined by a large effect size. This effect likely did not reach statistical significance due to the limited sample size (CG: *n* = 6, IG: *n* = 4).

No other changes of lateralized resting-state or task-based EEG activity were found in the intervention or the control group. Additionally, we did not find significant relationships between alpha asymmetry changes and our disorder-specific behavioral measures.

For the intervention group, the observed increase in right-lateralized frontopolar activity during resting-state - and based on effect size, also during reappraisal generation- may reflect a partial normalization of previously reported right-hemispheric functional deficits in patients with FND. Prior neuroimaging studies have consistently described reduced activation in FND in right prefrontal areas, including the DLPFC and inferior frontal gyrus—regions implicated in cognitive control and emotion regulation (Aybek and Vuilleumier 2016). Similarly, Morris et al. (2017) found that impaired avoidance learning in FND was associated with reduced activation in prefrontal circuits, including the DLPFC. While our EEG data do not allow for precise localization of these subregions, the right-lateralized frontopolar asymmetry observed here is consistent with this broader pattern of dysfunction. Our findings suggest that cognitive reappraisal training enhances right-lateralized frontal functioning, which may be relevant for improved emotion regulation (also see Falquez et al. 2014; Ochsner et al. 2004) as well as the observed clinical improvement of patients post intervention.

Altogether, our pattern of EEG findings suggests the relevance of right frontal cortex regions for successful reappraisal implementation. For resting-state EEG, this seems to be at odds with the seminal motivational direction model (Davidson 1998; Harmon-Jones et al., 2010) linking more left-lateralized frontal activity at rest to more adaptive approach motivation, and more right-lateralized frontal activity to more maladaptive avoidance motivation. However, the neural substrates of cognitive reappraisal are likely more complex than this basic approach-avoidance dichotomy.

Independent from any training, higher cognitive reappraisal performance (fluency) at baseline was associated with increased right-hemispheric dorsolateral frontal activity at rest in both groups. This not only replicates previous links of right-lateralized frontal activity (albeit during reappraisal generation) with greater reappraisal success (see Perchtold-Stefan et al., 2024), but also supports the idea that right frontal regions may be relevant for active engagement in cognitive emotion regulation (see Ochsner et al. 2004).

In this regard, previous studies highlighted the relevance of right frontal cortex regions for specific cognitive reappraisal processes that may be particularly impaired in FND patients: While left frontal brain regions are believed to facilitate more general linguistic and semantic components in cognitive reappraisal, along with more cognitive flexibility in switching between various reappraisal ideas (Ochsner et al. 2004; Papousek et al. 2017), right frontal regions were linked to more specific executive demands, such as inhibiting negative affect and adopting a more neutral, third-person perspective to successfully implement reappraisal ideas (Aron et al. 2014; Falquez et al. 2014). The relevance of right frontal areas for emotional distancing nicely corresponds with our finding that the frontopolar activity change towards the right post-training correlated with higher use of the reappraisal strategy of relativizing. Per definition, this strategy requires detachment from emotional stimuli by reducing their personal relevance (Shiota and Levenson 2012; Perchtold-Stefan et al., 2024), which may be a particularly useful strategy for FND patients. In this regard, research suggests that distancing/relativizing may be more easily implemented, as it only requires a shift from negative to neutral appraisals, compared to other more demanding strategies like positive re-interpretation that require a shift from negative to positive appraisals (Shiota and Levenson 2012).

This interpretation is strengthened by our finding that general training performance in the app (reappraisal and reflection in both groups) correlated with increased right frontopolar resting-state activity post training, linking successful training performance to adaptive changes in brain activity and supporting cognitive reappraisal as a trainable skill (Perchtold-Stefan et al. 2024). While it may be seen as a limitation that these changes also occurred in the control group, a retrospective assessment suggests that a portion of the reflection exercises (e.g., Think about a small unexpected positive thing that happened today) may have also tapped into, and thus trained, cognitive reappraisal processes (see below). Still, reappraisal training performance in the app only positively correlated with post-training reappraisal fluency in the intervention group, suggesting broader benefits from specific cognitive reappraisal training.

Although in need of replication, both the right-lateralized EEG and the behavioral findings tentatively suggest that the implemented cognitive reappraisal training improved more specific attentional control and affective response inhibition in FND patients, which may have improved their reappraisal implementation in daily life (also see Powers and LaBar 2019).

Still, our study may have benefitted from also including moment-to-moment subjective ratings of emotional valence or cognitive reappraisal success, which may have offered additional insights into the dynamics of the cognitive reappraisal process. However, we are confident that our use of a validated psychometric performance test for cognitive reappraisal generation during EEG acquisition offers meaningful insight into individual task engagement and cognitive strategy use—factors that are central to the interpretation of training-related changes in brain activity.

While our intervention focused exclusively on cognitive reappraisal training and did not include psychoeducation about FND, the literature suggests that psychoeducation can support diagnosis acceptance, treatment engagement, and symptom understanding (Hillyer et al., 2025; Guy et al., 2024). Future studies should consider integrating structured psychoeducation to enhance patient outcomes and therapeutic impact.

With regard to EEG-based brain correlates of cognitive reappraisal, future studies should also consider investigating other frequency bands associated with affective changes and emotion regulation, in particular theta (see Abid et al. 2025; Ertl et al., 2013).

Regarding behavioral variables, the improvement in everyday functioning in both groups may partly be attributed to the increased attention and empathetic communication from healthcare providers. Effective provider-patient relationships are known to enhance outcomes and patient satisfaction (Falkenström et al. 2013). Additionally, the reflection tasks and the cognitive reappraisal training may have been fairly similar in nature, thereby reducing group differences by boosting more positive reappraisal and stress reduction in both groups (Bartos et al. 2022). More distinct control tasks, such as relaxation or non-verbal activities, should be considered in future designs in order to clarify the specific effects of cognitive reappraisal training.

The discrepancy between more clear results in the subjective/objective clinical scores vs. the motor scores may be explained by the broader sensitivity of the clinical scores to subjective experiences of symptom control, emotional regulation, and overall well-being, in contrast to the motor scores, which more narrowly target observable motor symptoms. Given that the intervention primarily aimed to enhance cognitive emotion regulation, perceived improvements may precede measurable motor changes in time. Additionally, subtle functional gains may not be fully captured by the FTM or S-FMDRS, and the limited sample size may have further constrained the detection of statistically significant motor effects.

While overall, health measures improved significantly after the interventions, subjective improvements as reported by patients were less pronounced, reflecting common discrepancies in clinical research due to diverging perceptions of progress (Kupper and Tschacher 2008). This highlights the relevance of our study approach that supplemented behavioral findings with specifically tailored EEG investigations that may reveal faster and more subtle changes in cognitive reappraisal processes. In a previous study, adaptive neuronal changes were shown to precede changes in self-reported health measures that may only occur through more time and repeated practice of reappraisal in daily life (Perchtold-Stefan et al. 2024).

A relevant feasibility concern relates to the dropout rate, with 8 of 26 patients (30.7%) discontinuing participation. Six withdrew before the first assessment due to the time-intensive protocol and difficulties scheduling four appointments (two EEG sessions and two clinical assessments) within four weeks. Two others dropped out after the first session and after completing some training sessions for personal reasons (one of them was diagnosed at the start of the second visit with an acute depressive episode that required inpatient psychiatric admission and one had an acute family emergency to attend to in another country). These insights underscore the need to reduce logistical burden in future studies, for example by consolidating assessments at a single clinical site to improve retention and to exclude patients with severe depression.

Methodological limitations, including small sample size and repeated use similar tasks may have reduced the sensitivity of our study protocol. Future studies should thus focus on larger sample sizes, as well as better balanced reappraisal tasks to follow-up on our reported effects (Papousek et al. 2017).

One patient with comorbid, mild, and stable Parkinson’s disease was included, as functional symptoms were clearly predominant and neurodegenerative disorders were not defined as an exclusion criterion in the original protocol. The patient was under stable treatment for PD, with functional symptoms clearly representing the main clinical presentation. In future studies, patients with neurodegenerative disorders should ideally be excluded or analyzed in separate groups. This is particularly important, as functional comorbidity is frequent in Parkinson’s disease and deserves systematic investigation, but not within mixed cohorts.

A further important limitation of the study is the lack of follow-up data to evaluate whether any observed effects are maintained over time. In the absence of longitudinal assessments, it remains unclear if the outcomes reported reflect persistent changes or short-term fluctuations. Future research should incorporate long-term follow-up to address this gap.

It also should be mentioned that while clinical raters were blinded to group allocation, they were not blinded to the timing of assessment (pre- vs. post-intervention). Although standardized rating scales were used and raters were unaware of the specific intervention the patients received, the lack of blinding to time introduces a potential source of bias. Future trials should consider video-based motor assessments to ensure blinding not only to treatment group but also to assessment time point, thereby enhancing the objectivity of clinical ratings. Despite these limitations, our cognitive reappraisal training shows promise for improving symptom management and quality of life in FMD patients. Though in need of future development, mobile app-based training approaches as implemented in the present study are practical and promising techniques to help reduce symptomatic burden in patients who currently lack access to more comprehensive therapy (Morello et al. 2023).

Given our small sample size, our present findings should be interpreted under consideration of their preliminary, pilot nature. Still, in contrast to other studies lacking control arms (e.g., Reid et al. 2022; Hebert et al. 2021), our study incorporated a methodologically robust design by use of an active control group that ensures equal levels of participation for all patients. This helps to isolate the specific effects of the intervention itself from general effects of study involvement, and thus nonspecific factors such as therapeutic attention (Nielsen et al. 2024). In light of these methodological strengths, the current findings provide a strong rationale for conducting larger-scale confirmatory trials to further investigate the efficacy and neural mechanisms of app-based cognitive reappraisal training in patients with FMD.

## Data Availability

The data that support the findings of this study are available from the corresponding author upon reasonable request.
